# Airway basal cells from human-induced pluripotent stem cells: a new frontier in cystic fibrosis research

**DOI:** 10.3389/fcell.2024.1336392

**Published:** 2024-04-26

**Authors:** Anna Demchenko, Lyubava Belova, Maxim Balyasin, Konstantin Kochergin-Nikitsky, Ekaterina Kondrateva, Ekaterina Voronina, Victoria Pozhitnova, Vyacheslav Tabakov, Diana Salikhova, Tatiana Bukharova, Dmitry Goldshtein, Elena Kondratyeva, Tatiana Kyian, Elena Amelina, Olga Zubkova, Olga Popova, Tatiana Ozharovskaia, Alexander Lavrov, Svetlana Smirnikhina

**Affiliations:** ^1^ Laboratory of Genome Editing, Research Centre for Medical Genetics, Moscow, Russia; ^2^ Scientific and Educational Resource Center, Peoples’ Friendship University of Russia, Moscow, Russia; ^3^ Department of Cell Technology, Endocrinology Research Center, Moscow, Russia; ^4^ Laboratory of Mutagenesis, Research Centre for Medical Genetics, Moscow, Russia; ^5^ Moscow Branch of the Biobank “All-Russian Collection of Biological Samples of Hereditary Diseases”, Research Centre for Medical Genetics, Moscow, Russia; ^6^ Stem Cell Genetics Laboratory, Research Centre for Medical Genetics, Moscow, Russia; ^7^ Scientific and Clinical Department of Cystic Fibrosis, Research Centre for Medical Genetics, Moscow, Russia; ^8^ Laboratory of Cystic Fibrosis, Research Institute of Pulmonology, Moscow, Russia; ^9^ Federal State Budget Institution “National Research Centre for Epidemiology and Microbiology Named After Honorary Academician N F Gamaleya” of the Ministry of Health of the Russian Federation, Moscow, Russia

**Keywords:** airway basal cells, disease modeling, induced pluripotent stem cells, differentiation, cystic fibrosis, cystic fibrosis transmembrane conductance regulator, gene therapy, viral vectors

## Abstract

Human-induced airway basal cells (hiBCs) derived from human-induced pluripotent stem cells (hiPSCs) offer a promising cell model for studying lung diseases, regenerative medicine, and developing new gene therapy methods. We analyzed existing differentiation protocols and proposed our own protocol for obtaining hiBCs, which involves step-by-step differentiation of hiPSCs into definitive endoderm, anterior foregut endoderm, NKX2.1+ lung progenitors, and cultivation on basal cell medium with subsequent cell sorting using the surface marker CD271 (NGFR). We derived hiBCs from two healthy cell lines and three cell lines with cystic fibrosis (CF). The obtained hiBCs, expressing basal cell markers (NGFR, KRT5, and TP63), could differentiate into lung organoids (LOs). We demonstrated that LOs derived from hiBCs can assess cystic fibrosis transmembrane conductance regulator (CFTR) channel function using the forskolin-induced swelling (FIS) assay. We also carried out non-viral (electroporation) and viral (recombinant adeno-associated virus (rAAV)) serotypes 6 and 9 and recombinant adenovirus (rAdV) serotype 5 transgene delivery to hiBCs and showed that rAAV serotype 6 is most effective against hiBCs, potentially applicable for gene therapy research.

## Introduction

Airway human-induced basal cells (hiBCs) are tissue-specific progenitor epithelial cells capable of self-renewal and multi-lineage differentiation into specialized cells of the lung (Rock et al., 2009). hiBCs have promising areas of use, such as lung disease modeling, high-throughput screening, the development of new methods of genetic therapy, and regenerative medicine (Yu et al., 2023). hiBCs are characterized by the expression of the following markers: tumor protein 63 (TP63), cytoskeletal protein keratin 5 (KRT5), and nerve growth factor receptor (NGFR) (Nakamura et al., 2002; Rock et al., 2010; Yu et al., 2023). The derivation of hiBCs from hiPSCs is a promising and convenient method since obtaining somatic cells (peripheral blood monocytes or skin fibroblasts) for subsequent reprogramming into hiPSCs is often a minimally invasive procedure compared to obtaining tissue-specific cells. Several methods of hiPSC differentiation into hiBCs have been described. Among them are the derivation of NK2 homeobox 1 positive (NKX2.1+) lung progenitors from hiPSCs, the formation of airway organoids, cell sorting for the surface marker(s) of airway basal cells, and the cultivation of the cells either in the form of spheroids in a medium with small molecules inhibitors of SMAD signaling (Hawkins et al., 2021; Ngan et al., 2022) or in 2D culture on a feeder layer (de Carvalho et al., 2019). Among other works, the authors did not enrich the population of hiBCs using cell sorting but cultured NKX2.1+ lung progenitors in a medium containing factors that induce the formation of hiBCs on the feeder layer (mitotically inactivated mouse 3T3-J2 cells) (Djidrovski et al., 2021) or without it (Wong et al., 2012; 2015). The use of the feeder layer is associated with a risk of contamination since the feeder layer can detach from the culture plastic during the removal of target cells (Llames et al., 2015), which is unacceptable for further clinical use (Zhang et al., 2013). In addition, the lack of enrichment of the hiBC population by cell sorting can lead to the production of a heterogeneous cell population and non-reproducibility from production to production, which can affect the results of experiments (Geraghty et al., 2014).

In this article, we described a protocol for obtaining hiBCs from hiPSCs, which has yet to be reported. We compared the efficiency of obtaining airway basal cells using cell sorting from NKX2.1+ lung progenitors or LOs with the surface markers of basal cells CD271 (NGFR). hiBCs are capable of forming lung organoids containing functional epithelial cells, which allows for analyzing the conductance of the cystic fibrosis transmembrane conductance regulator (CFTR) channel *in vitro*. We demonstrated the efficiency of non-viral and viral transgene delivery to hiBCs, which is important to develop new treatments, including gene therapy and genome editing.

## Materials and methods

### Cell culturing

The study was approved by the Ethics Committee of the Research Centre for Medical Genetics (Moscow, Russia) and conducted in accordance with the provisions of the Declaration of Helsinki of 1975. Patients and healthy donors signed informed written consent forms as anonymous participants in the study and donors of biological materials. Skin fibroblasts from two healthy donors (WT) and three cystic fibrosis patients with homozygous or heterozygous F508del mutations of the *CFTR* gene were used for reprogramming. The hiPSC lines used in this work are described in Table 1. Skin fibroblasts and hiPSCs were deposited and are now available at the Moscow Branch of the Biobank “All-Russian Collection of Biological Samples of Hereditary Diseases” (Research Center for Medical Genetics, Moscow, Russia). hiPSCs were maintained in the TeSR™-E8™ (STEMCELL Technologies, Canada) on a culture dish coated with Matrigel (Corning, USA).

**TABLE 1:** List of cell lines used in the research.

Donor	Diagnosis	*CFTR* mutation	hiPSC line	hiBC line	Reference
1	Cystic fibrosis	F508del/F508del	No1	No1	Kondrateva et al. (2020)
2	Cystic fibrosis	F508del/F508del	No2	No2	Kondrateva et al. (2021b)
3	Cystic fibrosis	F508del/W1282X	No3	No3	Kondrateva et al. (2021a)
4	Health	WT/WT	No4	No4	Salikhova et al. (2019)
5	Health	WT/WT	No5	No5	See Supplementary Material

Primary airway basal cells (primary BCs) were obtained from a human nasal biopsy. Brush biopsies were obtained from slightly changed mucous membranes during the period of remission of the disease using a disposable cytology brush with a working length of 1,150 mm and a diameter of 5.0 mm for a 2.0-mm channel BC-202D-5010 (Olympus Medical Systems Corp., Japan). Freshly isolated basal cells were cultured on a Matrigel-coated culture dish in basal cell media (BCM) consisting of PneumaCult™-Ex Plus Medium (STEMCELL Technologies, Canada) with 1 µM A83-01 (STEMCELL Technologies, Canada), 1 μM DMH1 (Sigma Aldrich, USA), 0.2 μM hydrocortisone (STEMCELL Technologies, Canada), and 100× penicillin–streptomycin (PanEco, Russia).

### Derivation of hiBCs from hiPSCs

hiBCs were obtained from NKX2.1+ lung progenitors or LOs. NKX2.1+ lung progenitors and LOs were derived from hiPSC lines No1–No5 by directed differentiation according to our previously published protocol (Demchenko et al., 2023). In brief, hiPSCs were differentiated into definitive endoderm (DE) cells using 100 ng/mL activin A (R&D Systems, USA), and 5 µM CHIR99021 (Tocris, UK). Then, DE cells were differentiated into anterior foregut endoderm (AFE) cells with 10 μM SB431542 (Tocris, UK) and 2 μM dorsomorphin (Tocris, UK) in the serum-free differentiation medium (SFDM) (SFDM: 75% IMDM (Thermo Fisher Scientific, USA), 25% Ham’s F12 (Thermo Fisher Scientific, USA), 100× B-27 (Thermo Fisher Scientific, USA), 200× N2 (Thermo Fisher Scientific, USA), 0.05% bovine serum albumin solution (Sigma Aldrich, USA), 0.45 mM 1-thioglycerol (Sigma Aldrich, USA), 100× GlutaMAX (Thermo Fisher Scientific, USA), 0.05 mg/ml L-ascorbic acid (Sigma Aldrich, USA), and 100 μg/mL primocin (InvivoGen, USA)). Then, the AFE was differentiated into NKX2.1+ lung progenitor cells with 3 µM CHIR99021, 10 ng/mL BMP4 (R&D Systems, USA), and 100 nM retinoic acid (Sigma Aldrich, USA) in SFDM. For obtaining LOs from NKX2.1+ lung progenitors, cells were placed in Matrigel and cultured in SFDM with 10 ng/mL FGF7 (R&D Systems, USA), 10 ng/mL FGF10, 10 ng/mL EGF (R&D Systems, USA), and 3 µM CHIR99021.

For obtaining hiBCs from NKX2.1+ lung progenitors, cells were cultured for at least 5 days on a Matrigel-coated culture dish in BCM. Then, the cells were harvested with the Versene solution (PanEco, Russia) and stained with anti-CD271 (BioLegend, USA) antibodies according to the manufacturer’s protocols. Cells were sorted using an S3e Cell Sorter (Bio-Rad, USA) in the SFDM medium supplemented with 10 μM Y-27632 (STEMCELL Technologies, Canada). The sorted cell suspensions were centrifuged at 150 g for 5 min and passaged on a Matrigel-coated culture dish in the BCM with 10 μM Y-27632. After 24 h, the medium was replaced with the Y-27632-free medium.

For obtaining hiBCs from LOs, organoids were cultured for 6–16 days. Then, the organoids were dissociated into single cells with 0.05% trypsin-EDTA (PanEco, Russia). The obtained cells were stained with anti-CD271 (BioLegend, USA) and sorted as described above.

The visualization of the stages of differentiation was carried out using the Lionheart FX Automated Microscope (BioTek, USA).

### ALI culture of hiBCs

The differentiation of hiBCs into multiciliated cells was carried out using the air**–**liquid interface (ALI) culture method. For this purpose, 6.5-mm Transwells with a 0.4 μm pore (STEMCELL Technologies, Canada) were coated with 0.3 mg/mL type I collagen (Gibco, USA) diluted in 20 mM acetic acid. hiBC line No **2** at the fourth passage was seeded on transwells at a density of 30×10^**3**^ cells per insert and cultured in BCM. Once the cells reached a monolayer, the medium in the upper chamber was completely removed, and the medium in the lower chamber was switched to an ALI-culture medium. The medium for ALI culture consisted of PneumaCult™-ALI Medium (STEMCELL Technologies, Canada), 4 μg/mL heparin (STEMCELL Technologies, Canada), 9.6 μg/mL hydrocortisone, 1 μM DAPT (STEMCELL Technologies, Canada), 50 U/mL penicillin, and 50 μg/mL streptomycin. ALI cultivation was carried out for 36 days, with medium replacement every 48 h.

### RNA preparation and RT–PCR

Total RNA was isolated from hiBC line No3 on the fourth passage and from primary BCs on the second passage using the RNeasy kit (QIAGEN, Netherlands). cDNA was synthesized from 500 ng of total RNA using MMLV Reverse Transcriptase (Evrogen, Russia) and a mix of oligo (dT) and random primers, according to the manufacturer’s protocol. The housekeeping gene *B2M* was used as a positive control for the reaction. Then, 2 μL of cDNA was added to each 25 μL end-point PCR. PCR was performed using the gene-specific primers (Table 2), Taq polymerase (Evrogen, Russia), and the ProFlex PCR System (Applied Biosystems, USA). The cycling parameters were as follows: 95°C for 5 min, followed by 40 cycles of 20 s at 95°C, 5 s at 58°C, and 5 s at 72°C. The DNA electrophoresis of PCR products was performed in a 2% agarose gel and TBE buffer using Gel Loading Dye Blue (Evrogen, Russia) and a 100-bp + DNA ladder (Evrogen, Russia).

**TABLE 2:** List of RT–PCR primer sequences.

Target	Sequence (5’–3′)	Amplicon length (bp)
*TP63*	F: CAA​AGA​CAT​GCC​CCA​TCC​AG	197
	R: GCT​GTT​CCC​CTC​TAC​TCG​AA	
*KRT5*	F: CAG​TGG​AGA​AGG​AGT​TGG​ACC	146
	R: CAC​TGC​TAC​CTC​CGG​CAA​G	
*NGFR*	F: TGT​CTA​TTG​CTC​CAT​CCT​GGC	102
	R: CTG​TTG​GCT​CCT​TGC​TTG​TTC	
*B2M*	F: CTG​CCG​TGT​GAA​CCA​TGT​GA	103
	R: CAA​TCC​AAA​TGC​GGC​ATC​TTC	

### Karyotyping

hiBC line No2 at the fifth passage and approximately 80% confluence were arrested using 0.1 μg/mL demecolcine (Sigma Aldrich, USA), harvested by trypsinization, hypotonized for 13 min in 0.075 M KCl at 37°C, and fixed using standard cytogenetic procedures. Slides were stained with VECTASHIELD Mounting Medium with DAPI (Vector Laboratories, USA) contrasted with 0.3 mg/mL actinomycin D (SERVA, Germany). At least 15–20 metaphase images were analyzed according to ISCN 2020 nomenclature.

### Cell growth rate study

To study the cell growth rate, hiBC lines No1, No2, and No4 at the third passage were seeded at a density of 1×10^3^ cells per well (n = 3) on a 24-well plate in BCM. Phase-contrast images with a ×10 objective were captured using the Lionheart FX Automated Microscope every 24 h until the cells reached full confluence. Cell segmentation and analysis were performed using following open-source software: Cellpose 2.0 with human-in-the-loop iteration and the TN2 pre-trained model (Stringer et al., 2021; Pachitariu and Stringer, 2022) with subsequent CellProfiler 4.2.5 (Stirling et al., 2021) were used to calculate cells per field of view. The population doubling time (PDT) was calculated using the formula: PDT = T*ln2/ln (C2/C1), where T is the time of growth (days) and C1 and C2 represent the cell count per field of view at the start and end of growth, respectively.

### Derivation of LOs from hiBCs

For obtaining LOs from hiBC lines No1, No2 and No5, cells were harvested with the Versene solution, counted using a Countess II FL Automated Cell Counter (Thermo Fisher Scientific, USA), and centrifuged at 150× g for 5 min. The pellet was re-suspended in undiluted cold Matrigel at a concentration of 400–1,000 cells/μL and replated in 20 μL drops into the wells of a 48-well plate (Corning, USA). The drops were allowed to solidify for 40 min in an incubator, after which the SFDM supplemented with 10 ng/mL FGF7, 10 ng/mL FGF10, 10 ng/mL EGF, 3 µM CHIR99021, and 10 μM Y-27632 was added. After 24 h, the medium was replaced with the Y-27632-free medium.

### Forskolin-induced swelling of LOs derived from hiBCs

Forskolin-induced swelling (FIS) of organoids derived from hiBC lines No1, No2, and No5 was performed on day 7 or later after the passaging in Matrigel. The day before the analysis, the organoids were passaged into a 96-well plate with a droplet volume of 3 μL in SFDM with 10 ng/mL FGF7, 10 ng/mL FGF10, 10 ng/mL EGF, and 3 µM CHIR99021. On the day of the analysis, Calcein Green (Thermo Fisher Scientific, USA) was added to the wells with organoids at a final concentration of 0.25 μM and incubated for 40 min in an incubator. An image was taken on the Lionheart FX Automated Microscope. After that, forskolin (Sigma-Aldrich, USA) was added at a final concentration of 10 μM and incubated for 24 h, and an image was taken.

To estimate the swelling of organoids induced by forskolin, an FIS analysis was performed. The methodology for this analysis was described by Demchenko et al. (2023). The resulting images were analyzed using ilastik software v.1.3.3 (European Molecular Biology Laboratory) (Berg et al., 2019) and CellProfiler software v.4.2.1 (Carpenter et al., 2006). The area of the organoids was normalized, taking the area of each organoid at 0 h after the addition of forskolin as 100%.

### hiBC transfection

Transfections of hiBC line No4 were performed by electroporation using the Neon Transfection System (Thermo Fisher Scientific, USA). Cells were harvested with the Versene solution and re-suspended at a concentration of 3×10^3^ cells/μL in Opti-MEM (Thermo Fisher Scientific, USA) with 100× GlutaMAX (Thermo Fisher Scientific, USA) and 10 μM Y-27632 and 0.2 μg pEGFP-С1 plasmid (Clontech, USA) per 3×10^4^ cells. The cell suspension with plasmid was loaded into a 10 µL Neon Pipette Tip (Thermo Fisher Scientific, USA) and electroporated with two consecutive pulses at 1290 V for durations of 20 msec. Then, cells were placed into a culture dish with BCM with 10% fetal bovine serum (FBS) and 10 μM Y-27632 (without antibiotics). After 24 h, the medium was replaced with BCM without 10% FBS and 10 μM Y-27632 (with antibiotics). The visualization of the cell fluorescence was carried out using the Lionheart FX Automated Microscope. Transfection efficiencies were assessed 48 h after infection by flow cytometry using a CytoFLEX S (Beckman Coulter, USA).

### hiBC transduction

Transductions of hiBC line No4 were performed using rAAV serotypes 6 and 9 carrying the green fluorescent protein (GFP) transgene (rAAV2/6-GFP and rAAV2/9-GFP, respectively) and rAdV serotype 5 carrying the red fluorescent protein (mCherry) transgene (rAdV5-mCherry). The serotypes of the viral vectors we selected have a tropism for respiratory epithelial cells (Lenman, 2016; Belova et al., 2023). The plasmid pAAV-CMV-GFP was a gift from Dr. S. P. Chumakov from the Institute of Bioorganic Chemistry (Moscow, Russia); pAAV2/9 was a gift from Dr. James M. Wilson (Addgene plasmid #112865; http://n2t.net/addgene: 112,865; RRID: Addgene 112,865); pAAV2/6 was purchased from Takara Bio (#6651, Japan). The protocol for the generation of rAAV was previously described (Hild and Jaffe, 2016).

rAdV was generated using a traditional approach. First, an mCherry was cloned into the pShuttle plasmid, resulting in pShuttle-mCherry, where the mCherry reporter gene was placed under the control of a cytomegalovirus (CMV) immediate early promoter. Second, we generated a recombinant plasmid pAdV5-mCherry via the recombination of the backbone vector, containing most of the adenoviral genome, and the plasmid pShuttle-mCherry in *Escherichia coli* strain BJ5183, according to the standard protocol. Finally, the recombinant virus rAdV5-mCherry was obtained via lipofection in HEK293 cells using Lipofectamine 2000, according to the manufacturer’s protocol (Thermo Fisher Scientific, MA, USA). Adenovirus-producing cells were detected under fluorescent microscopy as fluorescent comet-like foci. For virus amplification, HEK293 cells were cultured in ten 150-mm cultural dishes. When cells grew to approximately 80%–90% confluence, we infected the cells with rAdV5-mCherry. After a full cytopathic effect (CPE) occurred, rAdV5-mCherry was purified from crude lysates of the monolayer culture using double cesium chloride gradient centrifugation. The titer of rAdV5-mCherry was determined using a plaque assay on HEK293 cell culture.

Transductions of the hiBC third–sixth passage were performed at 50% cell confluency in 24-well plates with 1×10^5^ cells per well. Transduction by rAAV was conducted with a multiplicity of infection (MOI) from 5E + 02 to 5E + 06 vg/cell in BCM. Transduction by rAdV5 was conducted with a plaque-forming unit (pfu) from 1 to 100 in BCM. All experiments were performed in three biological and three technical replicates. After 24 h, the media were changed. The visualization of the cell fluorescence was carried out using the Lionheart FX Automated Microscope. Transduction efficiencies were assessed 48 h after infection by flow cytometry using a CytoFLEX S.

### Immunofluorescence assay

hiBCs were immunostained on NKX2.1, KRT5, and TP63. Primary BCs were used as a positive control group. Also, hiBCs were stained onto CFTR protein for demonstrating protein expression. For immunostaining of hiBC lines No1, No2, and No4 at the fifth passage and primary BCs at the second passage, cells were grown on a Matrigel-coated 48-well plate. Cells were washed twice with DPBS (PanEco, Russia) and fixed with 4% paraformaldehyde (PFA) (Carl Roth, Germany) in PBS for 10 min at 37°C. The cells were permeabilized in a cold solution of 0.1% Tween 20 (Merck, Germany) for 10 min at +4°C and washed three times with DPBS; then cells were blocked with a cold solution of 0.1% Triton X-100 (Helicon, Russia) and 0.2% bovine serum albumin (BSA) (Sigma Aldrich, USA) in DPBS for 30 min at room temperature (RT: 20°C–25°C). Primary antibodies were added (Table 3) and incubated for 1 h at RT. Following this, the cells were washed three times with DPBS. Then, secondary antibodies were added (Table 3) and incubated for 30 min at RT; the cells were washed three times with DPBS. After that, cells were stained with DAPI (Abcam, UK) and visualized using the Lionheart FX Automated Microscope. Morphometric analysis was performed to assess the percentage of positive cells (KRT5+ and TP63+) and double-positive cells (NKX2.1+/KRT5+ and NKX2.1+/TP63+). Images (five images for each marker) were processed and analyzed using open-source software CellProfiler version 3.0.0. Cell nuclei and cytoplasm were determined, after which the average fluorescence intensity of staining per cell was measured, and the percentage of positive cells in the green and red channels was counted.

**TABLE 3:** Antibodies used in the immunofluorescence assay.

Antibody	Concentration
KRT5 (ABclonal, USA)	6 μg/mL
TP63 (Thermo Fisher Scientific, USA)	10 μg/mL
NKX2.1 (Thermo Fisher Scientific, USA)	9.8 μg/mL
CFTR (Abcam, UK)	0.4 μg/mL
FOXJ (Abcam, UK)	1 μg/mL
β-tubulin (Affinity Biosciences, China)	0.5 μg/mL
Muc5AC (Thermo Fisher Scientific, USA)	4 μg/mL
SCGB3A2 (Abcam, UK)	10 μg/mL
AQP1 (Sigma-Aldrich, USA)	1.5 μg/mL
SFTPB (Thermo Fisher Scientific, USA)	20 μg/mL
Goat Anti-Rabbit IgG H&L (Alexa Fluor 488) (Abcam, UK)	20 μg/mL
Goat Anti-Mouse IgG H&L (Alexa Fluor 488) (Abcam, UK)	20 μg/mL
Goat Anti-Rabbit IgG H&L (Alexa Fluor 594) (Abcam, UK)	20 μg/mL

To confirm the differentiation of hiBC line No2 into multiciliated cells, immunofluorescent staining was performed for markers of ciliated cells—β-tubulin and forkhead box protein J1 (FOXJ1). For this purpose, ALI cultures were washed twice with DPBS and fixed with 4% PFA in DPBS for 10 min at 37°C. The cells were permeabilized in a cold solution of 0.1% Tween 20 for 10 min at +4 °C and washed three times with DPBS; then cells were blocked with a cold solution of 0.1% Triton X-100% and 0.2% BSA in DPBS for 30 min at room temperature (RT: 20°C–25°C). Primary antibodies were added (Table 3) and incubated for 1 h at RT. Following this, the cells were washed three times with DPBS. Then, secondary antibodies were added (Table 3) and incubated for 30 min at RT; the cells were washed three times with DPBS. After that, cells were stained with DAPI. Then, membranes from Transwell were placed in a solution of 2.5 mM fructose (Sigma-Aldrich, USA) in 60% glycerol (PanReac AppliChem, Spain) and incubated for 20 min at room temperature. The suspension was transferred onto a glass slide (Pyrex, France) and covered with a cover glass (Pyrex, France); microscopy was performed on a TCS SP8 confocal laser scanning microscope (Leica Microsystems, Germany).

Immunostaining of LOs on 7 days of differentiation from hiBCs was performed according to the protocols of Dekkers et al. (2019). In brief, droplets with organoids were mechanically dislodged, centrifuged for 5 s at 6,300 g, and fixed with 4% PFA in DPBS for 45 min at +4 °C. Then, the organoids were permeabilized in a cold solution of 0.1% Tween 20 (Merck, Germany) for 10 min at +4 °C and centrifuged for 5 min at 70 g at +4°C; the precipitate was blocked with a cold solution of 0.1% Triton X-100% and 0.2% BSA in DPBS for 15 min at +4 °C. Then, a solution of primary antibodies was added, and the mixture was incubated overnight at +4°C (Table 3). The organoids were washed twice with a solution of 0.1% Triton X-100% and 0.2% BSA in DPBS without Ca^2+^ and Mg^2+^ for 2 h at +4 °C. Then, a solution of the secondary antibodies was added, and the mixture was incubated overnight at +4°C (Table 3). The organoids were washed twice with a solution of 0.1% Triton X-100% and 0.2% BSA in DPBS for 2 h at +4°C. After that, the organoids were stained with DAPI for 10 min at room temperature and subsequently centrifuged for 5 s at 6,300 g; the pellet was re-suspended in a solution of 2.5 mM fructose in 60% glycerol and incubated for 20 min at room temperature. The suspension was transferred onto a glass slide and covered with a cover glass; microscopy was performed on a TCS SP8 confocal laser scanning microscope (Leica Microsystems, Germany).

### Statistical data analysis

Statistical analysis of the data was performed using GraphPad Prism v.9.1.1. To compare the percentage of positive cells obtained by flow cytometry, a t-test was used. To compare the swelling area of lung organoids, Kruskal–Wallis tests with post-hoc comparisons using the Dunn’s method were used. Transduction and transfection efficiency were evaluated with a one-way ANOVA with post-hoc comparisons using the Tukey’s method. Data were considered significant if the *p*-value was <0.05.

## Results

### Derivation of hiBCs from hiPSCs

**FIGURE 1:**
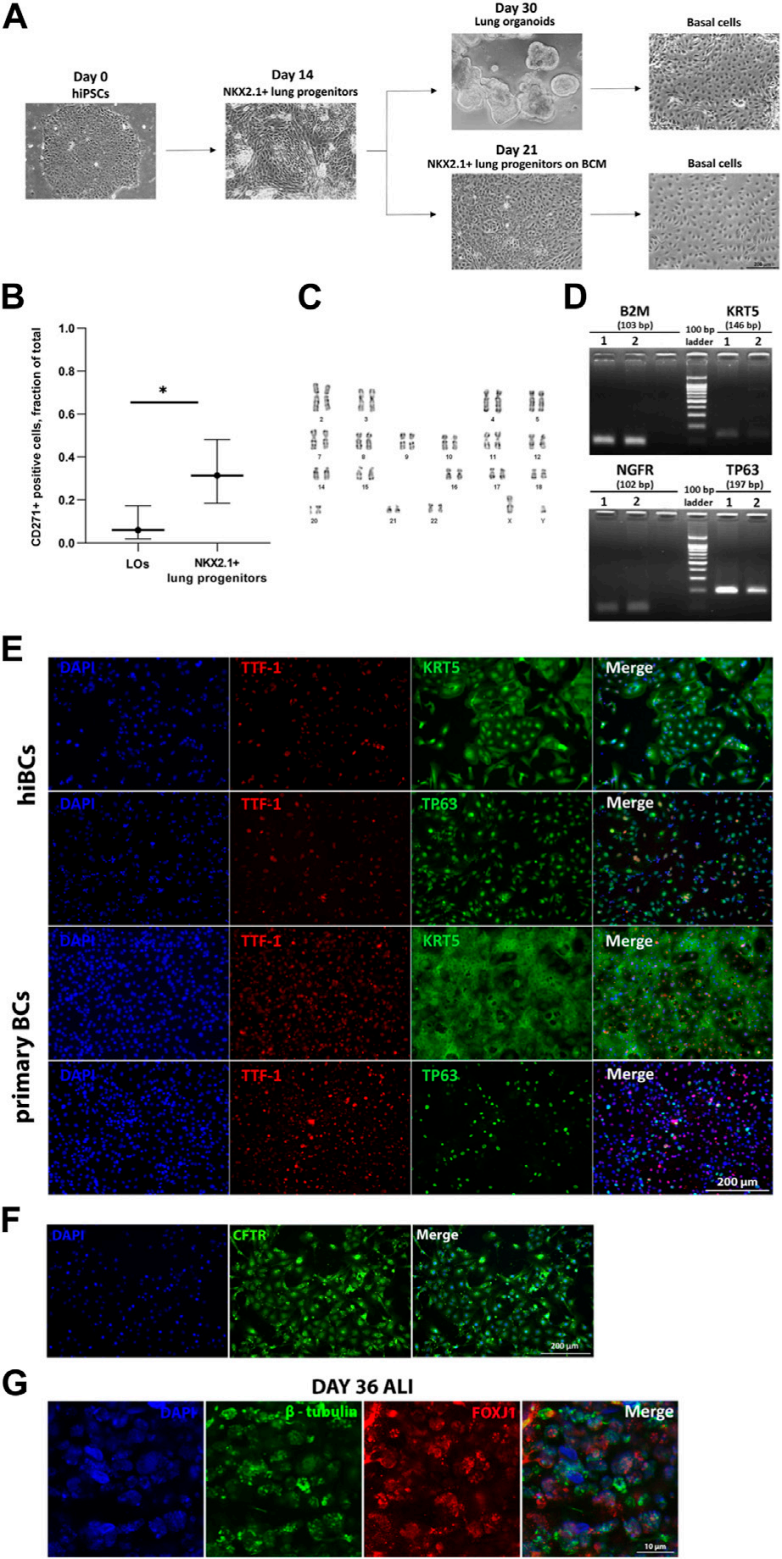
Derivation of hiBCs from hiPSCs and their characterization. **(A)** Representative phase-contrast images of each stage of differentiation of hiPSCs into hiBCs. Scale bar, 200 μm. **(B)** Flow cytometry assessment of CD271 expression in LOs and NKX2.1+ lung progenitors derived from two with homozygous F508del mutation in the *CFTR* gene. Data are presented as the median value ±95% CI, t-test, * — *p* < 0.03. **(C)** Karyotype of hiBCs derived from hiPSCs with the homozygous F508del mutation. **(D)** Qualitative assessment of the expression of cell markers in primary BC 1 and hiBC line No3 2 by RT-PCR. Size markers are indicated by horizontal lines. **(E)** Representative images from fluorescent microscopy of hiBC No2 stained against major basal cells markers compared to primary BCs obtained from a nasal biopsy of a healthy donor. Scale bar, 200 μm. **(F)** Representative images from fluorescent microscopy of hiBC No4 stained against CFTR antibody. Scale bar, 200 μm. **(G)** Representative images from fluorescent microscopy of 36-day ALI culture of hiBC No2 stained against marker of ciliary cells (β-tubulin and FOXJ1). Scale bar, 10 μm.

We differentiated five hiPSC lines into hiBCs: two lines from healthy donors (No4 and No5) and three lines from cystic fibrosis patients with homozygous (No1 and No2) or heterozygous (No3) F508del mutations of the *CFTR* gene. We obtained hiBCs by cell sorting on surface markers of basal cells CD271 (NGFR) from NKX2.1+ lung progenitors, after culturing on BCM, or from LOs (Figure 1A). The percentage of cells with the CD271 marker expression was 31.4% ± 0.2% (95% CI: n = 19) for NKX2.1+ lung progenitors and 6.1% ± 0.1% (95% CI: n = 5) for LOs, with statistically significant differences (t-test: *p* = 0.0201) (Figure 1B). Based on these results, we conclude that deriving hiBCs from NKX2.1+ lung progenitors is more efficient than from LOs. The evaluation of CD271+ cells in the hiBC fifth passage revealed that 95.2% ± 0.98% of the cells were positively stained. hiBCs are stably subcultured and maintained in culture (we cultivated cells up to the 10th passage; data not shown). The cells could also be frozen at 1 million per vial in 90% FBS and 10% dimethyl sulfoxide (DMSO) and stored in liquid nitrogen for later use. The PDT at the third passage of three lines of hiBCs was 3.5 ± 1.8 days (SD; n = 3). At the fifth passage, the line of hiBC line No2 showed a normal 46, XY karyotype (Figure 1C). hiBCs, as well as primary BCs, expressed major markers of basal cells (TP63, KRT5, and NGFR), as demonstrated in the electropherogram of RT-PCR products (Figure 1D). Furthermore, according to the results of immunofluorescence staining, positive staining of hiBCs and the control group (primary BCs) against antibodies to TP63, KTR5, and NKX2.1 is observed (Figure 1E). In hiBCs, the average percentage of TP63+ cells was 98.6% ± 2.6% and that of KRT5+ cells was 98.1% ± 3.5% (SD; n = 3 biological replicates from independent experiments). The percentage of NKX2.1+/TP63+ double-positive cells ranges from 96.13% to 99.95% and that of NKX2.1+/KRT5+ cells ranges from 97.1% to 99.84%. In primary BCs, the average percentage of TP63+ cells was 80.5% ± 11.3% and that of KRT5+ cells was 97.1% ± 1.8% (SD; n = 1 biological replicates from independent experiments). In addition, hiBCs derived from healthy donors (hiPSC No4) express CFTR (Figure 1F), which makes it possible to analyze changes in protein localization when genetically editing cystic fibrosis mutations in hiBCs (Carvalho-Oliveira et al., 2004). hiBCs have the ability to differentiate in the multiciliated direction. Staining on day 36 of ALI culture showed positive staining for the markers of ciliary cells: β-tubulin and FOXJ1 (Figure 1G).

### FIS of LOs derived from hiBCs

**FIGURE 2:**
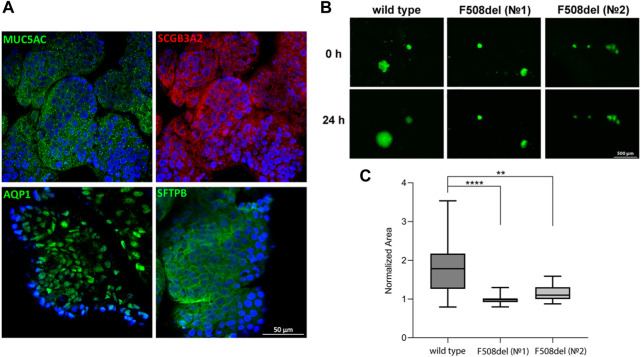
Cellular composition and FIS in LOs derived from hiBCs. **(A)** Representative images from confocal microscopy of LOs stained against major lung epithelial cell markers at 8 days of differentiation from hiBCs. Nuclei were stained with DAPI (blue). Scale bar, 50 μm. **(B)** Representative images of wild-type (cell line No5) and F508del (cell lines No1 and No2) LOs before and after 24 h of stimulation with forskolin (10 µM). Scale bar, 500 μm. **(C)** Quantification of the normalized (by time point 0) swelling area of lung organoids derived from wild-type and F508del hiBCs at time = 0 and 24 h. Kruskal–Wallis with post-hoc Dunn; Data are presented as the median ±95% CI; n = 2 biological replicates from independent experiments. The number of analyzed organoids in each group is 20–110 individual organoids. **—p < 0.0021 and ****—p < 0.0001.

Airway basal cells are the progenitor cells of the respiratory tract, so they are capable of differentiating into specialized cells. We derived LOs from one hiBC line from a healthy donor (line No5, wild type) and two hiBC lines from donors with cystic fibrosis with a homozygous mutation in *CFTR* (F508del) (lines No1 and No2, F508del). The LOs stained positively against major lung epithelial cell markers (MUC5AC, SCGB3A2, AQP1, and SFTPB), confirming the completion of the differentiation of hiBCs into LOs (Figure 2A). For functional analysis of the conductivity of the CFTR channel in organoids, an FIS assay of LOs was performed (Figure 2B). LOs derived from hiBC line No5 from a healthy donor swelled by 24 h 1.8 times (95% CI: 1.5–2.2), relative to the time point of 0 h. LOs derived from No1 and No2 line hiBCs with the F508del mutation in CFTR swelled by 24 h 0.9 times (95% CI: 0.9–1) and 1.2 times (95% CI: 1.1–1.2), respectively, relative to the time point of 0 h (Figure 2C). The obtained results confirm that hiBCs are capable of forming LOs with functional epithelial cells, which allows one to analyze the activity of the CFTR channel *in vitro*.

### Transfection and transduction of hiBC

**FIGURE 3:**
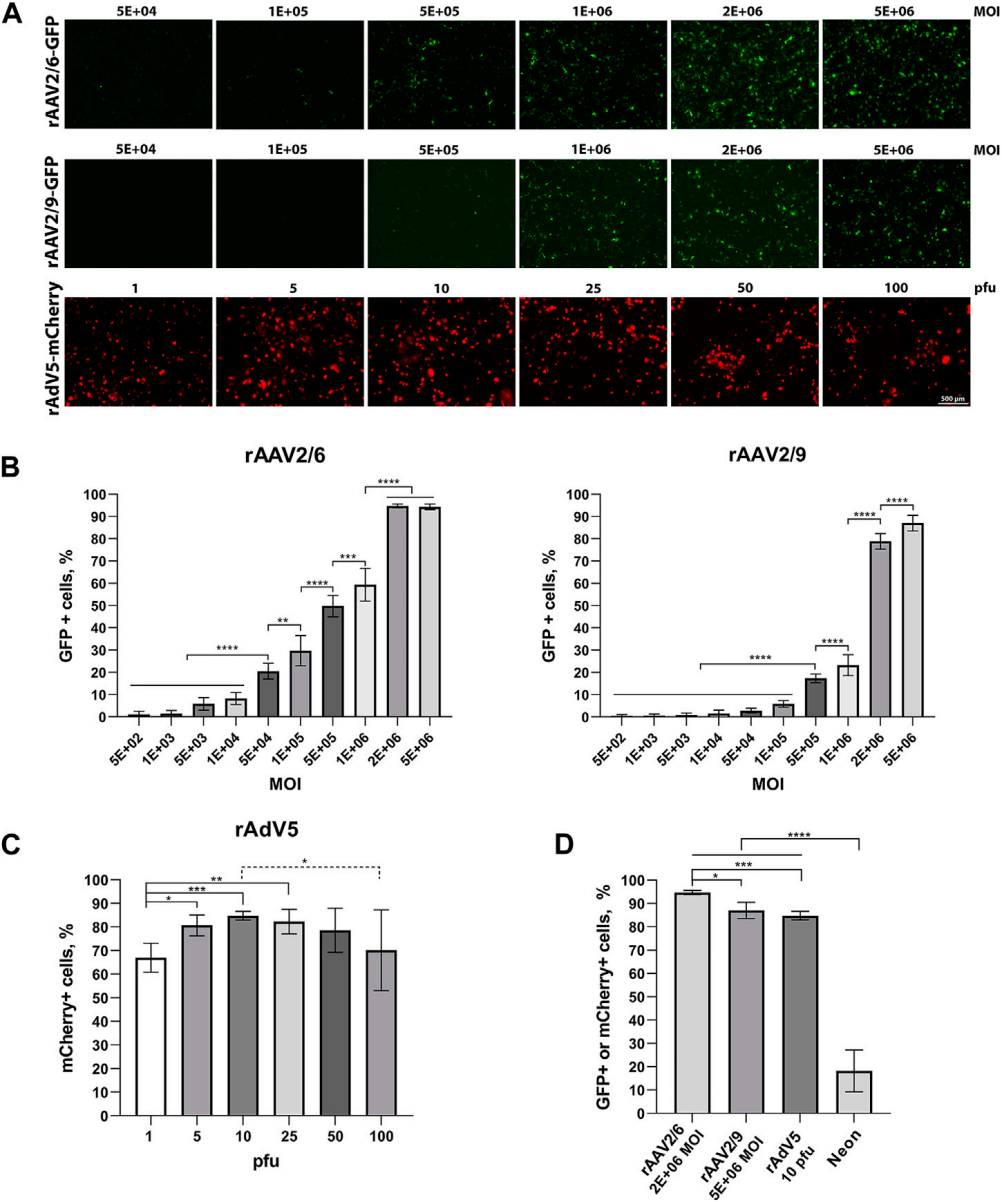
Transduction and transfection efficiency in hiBCs. **(A)** Representative images of GFP or mCherry fluorescence in hiBCs at 48 h after transduction using rAAV2/6, rAAV2/9, and rAdV5. Scale bar, 50 μm. **(B)** Transduction efficiency of rAAV2/6 and rAAV2/9. **(C)** Transduction efficiency of rAdV5. **(D)** Compare the maximum efficiency values of rAAV2/6, rAAV2/9, and rAdV5 transduction and transfection using Neon. In all graphs, data are presented as the mean ± SD; n = 3 biological replicates from independent experiments. ANOVA with post-hoc Tukey; *—p < 0.0332, **—p < 0.0021, ***—p < 0.0002, and ****—p < 0.0001.

hiBC line No4 was transduced with three viral vectors (rAAV2/6-GFP, rAAV2/9-GFP, and rAdV5-mCherry) and electroporated using the Neon with the pEGFP-C1 plasmid. The visualization of cell fluorescence at 48 h after transduction is shown in Figure 3A. The images did not show rAAV from 5E+02 to 1E+04 because the fluorescent signal was not detected using the microscope. The images of co-expression basal cell markers (TP63 and KRT) and transgenes (GFP and mCherry) are presented in Supplementary Figure S2. The maximum transduction efficiency at 48 h for rAAV2/6 and rAAV2/9 was 94.7% ± 0.9% at 2E+06 MOI and 87.1% ± 3.5% (SD) at 5E+06 MOI, respectively (Figure 3B). The maximum transduction efficiency at 48 h for rAdV5 was 84.8% ± 1.8% (SD) at 10 pfu (Figure 3C). The transduction efficiency at 48 h for rAdV5 at 25 pfu [82.3% ± 5.2%; (SD)] and 50 pfu [78.6% ± 9.4%; (SD)] was not statistically different from 10 pfu (post-hoc Tukey; *p* = 0.9890 and *p* = 0.6486, respectively); however, using 10 pfu as the optimal virus concentration allows for reduced vector dosing and a reduction in toxicity in future gene editing experiments. For example, the efficiency of plasmid transfection using Neon for 48 h was 18.2% ± 8.9% (SD). Thus, when comparing the efficiencies between the maximum values of rAAV2/6, rAAV2/9 rAdV5, and Neon electroporation, rAAV2/6 showed the best result with a statistically significant difference (ANOVA; *p*-value <0.05) compared to other methods of plasmid delivery (Figure 3D). At 48 h and 7 days after transgene injection, hiBCs demonstrated the preservation of basal cell morphology and the ability to form lung organoids at 7 days (Supplementary Figure S3).

## Discussion

In this study, we describe the differentiation of hiPSCs into hiBCs by sorting NKX2.1+ lung progenitors after culturing in BCM for at least 5 days on the basal cell surface marker CD271. The average efficiency of CD271+ cells was 31.4%, which is significantly higher (t-test; *p* = 0.0201) than isolating a population of hiBCs from the LOs. Our protocol is a bit similar to the previously published protocols by Wong A. P. et al., where the authors differentiated hiPSCs into NKX2.1+ lung progenitors and then cultivated the cells in a BCM (Wong et al., 2012; 2015). However, we believe that sorting for the surface marker of basal cells to enrich the hiBC population is necessary to ensure a robust protocol that produces a homogeneous culture of hiBCs from different donors. This is important for the future application of this cell model in the screening of therapeutic compounds aimed at correcting the CFTR mutation and for the development of etiotropic therapy for cystic fibrosis. In our work, we did not try to obtain hiBCs from AOs, as described in a number of works (Hawkins et al., 2021; Ngan et al., 2022), because AOs practically do not survive after cryopreservation, making them an inconvenient choice for experimentation. The hiBCs generated in this study expressed the main airway basal markers (NGFR, KRT5, and TP63) and lung progenitor cell marker NKX2.1 with a high proportion of double-positive cells of NKX2.1/KRT5 and NKX2.1/TP63, which confirms that hiBCs have the airway system origin. However, our findings show that KTR5 is localized to both the nucleus and cytoplasm in hiBC staining. In a study investigating the localization of KTR17, the authors found a correlation between the nuclear localization of KRT17 and the proliferative activity of cells (Jacob et al., 2020). We can only speculate on this topic, link the nuclear localization and good proliferative potential of hiBCs, and continue to study this fact. hiBCs were maintained in culture for more than 10 passages without changes in morphology. A similar number of passages is described in articles where hiBCs were cultured in the form of spheroids (8–10 passages) (Hawkins et al., 2021; Ngan et al., 2022) or on the feeder layer (at least 8 passages) (Djidrovski et al., 2021). The ability of hiBCs to be maintained in culture for a long time, as well as successfully cryopreserved without changing cell phenotype, makes cells a perspective cell model in cell biology.

For the first time, we showed the possibility of obtaining LOs from hiPSC-derived basal cells. Other studies have reported the derivation of spheroids from basal cells (Hawkins et al., 2021; Ngan et al., 2022) or bronchospheroids from primary airway basal cells (Hild and Jaffe, 2016; Pat et al., 2022). LOs are promising for use in FIS analysis to evaluate the efficacy of CFTR modulators for rare CFTR mutations. Previously, we demonstrated that FIS can be carried out on LOs derived from hiPSCs (Demchenko et al., 2023). In this article, we demonstrate the functional activity of the CFTR channel by FIS in LOs derived from hiBCs. The FIS assay on LOs derived from hiBCs can be used for diagnostic purposes to select targeted therapy for patients with CF, as is now generally accepted for intestinal organoids (Dekkers et al., 2013). However, CF is primarily a respiratory and digestive disease; the presence of a cellular model with tissue-specific properties is preferred. Intestinal organoids derived from rectal epithelium are not associated with CF pathology, raising questions about how representative this cellular model is of its respiratory counterpart (Clancy et al., 2019). We believe that obtaining LOs from hiBCs is much more convenient than direct differentiation from hiPSCs. hiBCs have a good recovery after cryopreservation, so having a biobank of hiPSC-derived hiBCs from CF patients can quickly and efficiently obtain LOs. We demonstrated that LOs from a patient with the homozygous F508del mutation practically did not swell after 24 h in response to forskolin, whereas healthy lung organoids swelled by 1.8 times.

In our work, we assessed the efficiency of transduction of rAAV2/6, rAAV2/9, and rAdV5 and transfection using the Neon Transfection System into hiBCs. The serotypes of the viral vectors have a tropism for lung epithelial cells (Lenman, 2016; Kochergin-Nikitsky et al., 2021). By comparing the maximum efficacy values in each group, we demonstrated that among rAAV vectors, rAAV2/6 is more effective for hiBC transduction (94.7% ± 0.9% at 2E+06 MOI) than rAAV2/9 (87.1% ± 3.5% at 5E+06 MOI, *p* < 0.0332). Furthermore, rAAV2/6 is more effective than rAdV5 (84.8% ± 1.8% at 10 pfu, *p* < 0.0002). Both types of viral vectors have their advantages and disadvantages. rAAV vectors have low immunogenicity and are non-pathogenic but have a limited capacity for gene insertion, while rAdV vectors, on the contrary, have a high DNA-packing capacity but high immunogenicity (Hild and Jaffe, 2016; McClements and MacLaren, 2017).

The transfection method using the Neon showed poor results compared to transduction (18.2% ± 8.9%, *p* < 0.0001). According to our results, the most efficient delivery of transgenes is by rAAV2/6, which could potentially be used for gene delivery to hiBC for gene therapy or genome editing. Since hiBCs are progenitor cells of the respiratory system, gene modifications of hiBCs will result in a longer-lasting therapeutic effect than modifications in more differentiated airway epithelial cells. We did not find any articles describing the transfection or transduction of hiBCs derived from hiPSCs, and therefore, we can only compare our results with primary cultures of human hiBCs. In Rapiteanu et al. (2020), transfection was carried out using an Amaxa 4D Nucleofector (Lonza). The efficiency of electroporation with subsequent gene editing was over 90%. The results we obtained are significantly lower, possibly due to differences in the electroporator and cell culture. In Li et al. (2019), the transduction of well-differentiated primary cultures of human airway epithelial cells (>3 weeks old at the ALI culture) of 14 AdV serotypes was carried out. AdV5 demonstrated low efficiency at 50 MOI for 4 h compared to AdV3 and AdV69, despite the fact that AdV5, like AdV3, is derived from species C, which efficiently transduces the respiratory tract, unlike AdV69, which is derived from species D and has tropism for the eye and intestine (Lenman, 2016). There are no published studies on AAV transduction of primary or induced basal cells with AAV, but it is worth noting that AAV6 was able to rapidly diffuse through mucus collected from CF patients, which also makes AAV6 a good candidate for the development of CF therapy (Duncan et al., 2018).

To summarize, we described in detail simplified protocols for the derivation of hiBCs from hiPSCs. We demonstrated, for the first time, that basal cells have the ability to differentiate into LOs with functionally active CFTR channels in healthy organoids. We also carried out non-viral and viral transgene delivery to hiBCs and showed that rAAV2/6 was the most effective against airway basal cells, which may be applicable to the delivery of therapeutic genes for gene therapy.

## Data Availability

The raw data supporting the conclusion of this article will be made available by the authors, without undue reservation.
