# Supplementary material for: Airway basal cells from human-induced pluripotent stem cells: a new frontier in cystic fibrosis research
Source: Front Cell Dev Biol. 2024 Apr 26;12:1336392. doi: 10.3389/fcell.2024.1336392 (PMC11082282; doi:10.3389/fcell.2024.1336392)
Supplement: Supplementary file 1 [file Table1.docx]

Supplementary Material

Airway Basal Cells from human-induced pluripotent stem cells: A New Frontier in Cystic Fibrosis Research

**Anna Demchenko*, Lyubava Belova, Maxim Balyasin, Konstantin Kochergin-Nikitsky, Ekaterina Kondrateva, Ekaterina Voronina, Victoria Pozhitnova, Vyacheslav Tabakov, Diana Salikhova, Tatiana Bukharova, Dmitry Goldshtein, Elena Kondratyeva, Tatiana Kyian, Elena Amelina, Olga Zubkova, Olga Popova, Tatiana Ozharovskaia, Alexander Lavrov and Svetlana Smirnikhina**

* Correspondence: [demchenkoann@yandex.ru](mailto:demchenkoann@yandex.ru)

**Generation and characterization of hiPSC line №5**

*hiPSCs derivation*

Fibroblasts were obtained from biopsy material using Amniocar proliferative medium (PanEco). Second passage was plated in fibroblast medium (DMEM, high glucose, GlutaMAX™ Supplement, pyruvate (Life Technologies), 1% MEM non-essential amino acids solution (Gibco), 50 U/ml penicillin, 50 ug/ml streptomycin, 10% fetal bovine serum (FBS)). Reprogramming of 100.000 cells/well was performed using ReproRNA™-OKSGM Kit and growth medium (Advanced DMEM (Gibco), 10% FBS, 2 mM L-glutamine) supplemented with 175 ng/ml B18R protein. Next day the cells were transferred to medium with 0.8 µg/mL puromycin for 6 days with daily replacing. On day 7, cells were transferred to B18R-contained medium without puromycin. On day 9, cells were transferred to ReproTeSR™ medium (Stemcell Technologies) with B18R. On the 15th day, the appearance of the first colonies was observed. On day 25, colonies were picked manually and replated into cell culture plates pre-coated with Vitronectin. The hiPSCs were cultured in Essential 8™ Medium (Gibco). GCDR was used to passage cells every 4-10 days depending on confluence. General ratio of split was 1:6. During every plating, Y27632 ROCK inhibitor was added at 5 μM into the medium for 24 hours. Cells were cultivated at 37°C, 5% CO2.

*Immunocytochemistry counting*

We fixed hiPSCs with 4% paraformaldehyde at 4°C for 20 minutes, permeabilized with 0,25% Triton X-100 in phosphate-buffered saline (PBS) at room temperature (RT: 20 to 25°C) for 10 minutes and blocked with 1% bovine serum albumin (BSA) at RT for 30 minutes. Incubation was performed with primary antibodies (Supplementary Table 1) in 1% BSA at 4°C overnight and with secondary antibodies at RT for 1 hour in the dark. Nuclei were counterstained using DAPI at RT for 10 minutes. Fluorescent images were captured using Lionheart FX Automated Microscope (BioTek). Cell nuclei and cytoplasm were determined, after which the average fluorescence intensity of staining per cell was measured and the percentage of positives cells in the green and red channels was counted.

**Supplementary Table 1.** Antibodies used for immunocytochemistry assay

|  | **Antibody** | **Dilution** |
| --- | --- | --- |
| Pluripotency Markers | SSEA4 Monoclonal Antibody (MC813-70) (Thermo Fisher Scientific, USA) | 1:100 |
|  | Anti-Oct4 antibody (Abcam, UK) | 1:100 |
|  | Nanog Polyclonal Antibody (Thermo Fisher Scientific, USA) | 1:100 |
|  | Anti-SOX2 antibody [9-9-3] (Abcam, UK) | 1:200 |
| Differentiation Markers | Anti-beta III Tubulin antibody [2G10] (Abcam, UK) | 1:300 |
|  | Anti-alpha 1 Fetoprotein antibody [AFP-01] (Abcam, UK) | 1:200 |
|  | Brachyury Monoclonal Antibody (X1AO2) (Thermo Fisher Scientific, USA) | 1:100 |
| Secondary antibodies | Goat Anti-Rabbit IgG H&L (Alexa Fluor® 647) (Abcam, UK) | 1:200 |
|  | Goat Anti-Mouse IgG H&L (Alexa Fluor® 488) (Abcam, UK) | 1:200 |

*Karyotyping*

At passage 15 GTG-banding analysis of at least 15 metaphase spreads was performed in-house based on ISCN 2016. Incubation in colchicine solution (PanEco) (10 ug/ml) lasted for 35-40 min to arrest mitosis followed by hypotonic treatment (0.075M KCl) at 37˚C for 13 min. Fixation was done by twice incubation in cooled solution made of 3 parts methanol and 1 part glacial acetic acid for 30 and 20 min.

*Spontaneous differentiation*

On the 12^th^ passage, the hiPSCs were detached using GCDR and transferred in E8 with Y27632 to ultra-low attachment plate (Corning Costar). On day 3 the half of medium was replaced and on the day 6 all the medium was replaced with medium consisting of Advanced DMEM/F12 (Gibco), 20% KO Serum replacement (Gibco), 1% MEM NEAA, 2 mM L-glutamine (Gibco), 50 U/ml penicillin, 50 ug/ml streptomycin. Thereafter, the medium was replaced every 2 days with an addition of 1% FBS and then stepwise increase it from 1 to 10%. In about 14 days embryoid bodies (EB) were transferred onto gelatin-coated plates in EB medium with 10% FBS, and after 14 more days EB immunocytochemistry was performed.

***Results***

Skin fibroblasts obtained from a 25-years-old healthy woman were reprogrammed into hiPSCs by using non-viral, non-integrating, self-replicating RNA reprogramming vector including five reprogramming factors: OCT4, KLF-4, SOX2, GLIS1, and c-MYC as well as a puromycin-resistance gene. hiPSC clone was purified (Supplementary [Figure 1](https://www.sciencedirect.com/science/article/pii/S1873506120300143#fig0001)A), analyzed and registered in hPSCreg database as RCMGi017-A. At 15^th^ passage the cells showed a normal 46, XX karyotype (Supplementary [Figure 1](https://www.sciencedirect.com/science/article/pii/S1873506120300143#fig0001)B).At 8^th^ passage the high level expression of pluripotency markers SSEA4, OCT4, SOX2, and NANOG was detected and counted by immunocytochemistry assay (Supplementary [Figure 1](https://www.sciencedirect.com/science/article/pii/S1873506120300143#fig0001)C, 1D). Potential of hiPSCs to differentiate into three germ layers was proven in spontaneous differentiation assay by formation of EB and analyzing them by immunocytochemistry staining which was positive for tubulin beta (ectodermal markers), brachyury (mesodermal markers), and alpha-1-fetoprotein (endodermal markers) (Supplementary [Figure 1](https://www.sciencedirect.com/science/article/pii/S1873506120300143#fig0001)E). Identical DNA profiles of the fibroblasts and the generated line were verified by short tandem repeat (STR) analysis of 20 microsatellite markers (STR report is available with the authors).


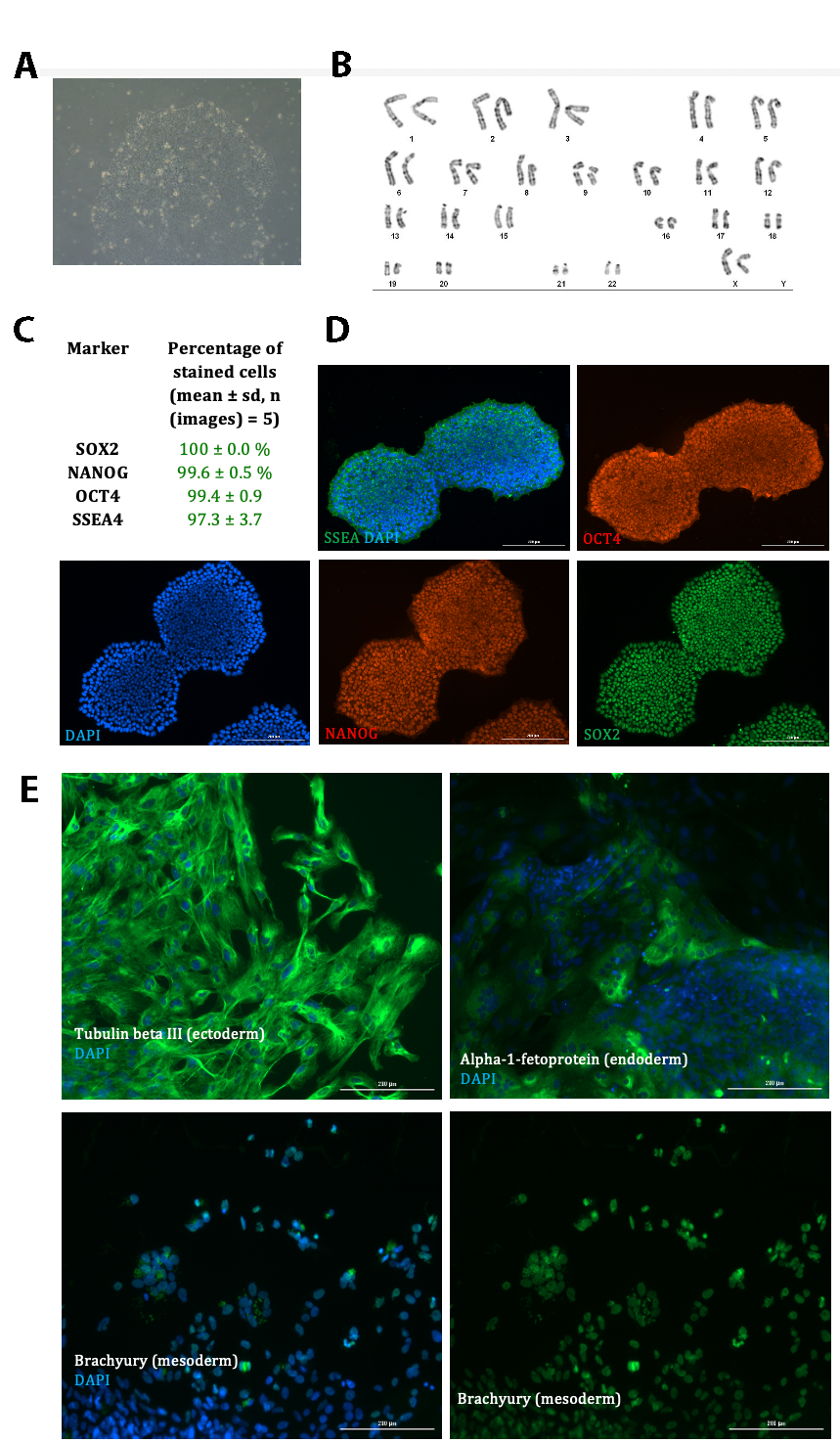


**Supplementary Figure 1.** Characterization of hiPSCs line №5

**Differentiation and maintenance of hiBC after electroporation and transduction**

*Immunofluorescence assay*

Immunofluorescence staining of hiBC line №4 48 h and 7 days after transduction for two basal cell markers (TP63 and KRT5) was performed. Cells were washed twice with DPBS and fixed with 4% paraformaldehyde (PFA), (Carl Roth, German) in PBS for 10 min at 37°C. The cells were permeabilized in a cold solution of 0.1% Tween 20 (Merck, Germany) for 10 min at +4 °C and washed three times with DPBS; then cells were blocked with a cold solution of 0.1% Triton X-100 (Helicon, Russia) and 0.2% bovine serum albumin (BSA, Sigma Aldrich, USA) in DPBS for 30 min at RT. Primary antibodies were added (Supplementary Table 2) and incubated for 1 h at RT. Following this, the cells were washed three times with DPBS. Then secondary antibodies were added (Supplementary Table 2) and incubated for 30 min at RT; the cells were washed three times with DPBS. After that, cells were stained with DAPI and visualized using the Lionheart FX Automated Microscope. Morphometric analysis was performed to assess the percentage of positive cells (KRT5+, TP63+). Images (5 images for each marker) were processed and analyzed using open-source software CellProfiler version 3.0.0. Cell nuclei and cytoplasm were determined, after which the average fluorescence intensity of staining per cell was measured and the percentage of positives cells in the green and red channels was counted.

Supplementary Table 2. Antibodies used in immunofluorescence assay

| **Antibody** | **Concentration** |
| --- | --- |
| KRT5 (ABclonal, USA) | 6 μg/ml |
| TP63 (Thermo Fisher Scientific, USA) | 10 μg/ml |
| GFP (Abcam, UK) | 5 μg/ml |
| mCherry (Abcam, UK) | 3 μg/ml |
| Goat Anti-Mouse IgG H&L (Alexa Fluor 488) (Abcam, UK) | 20 μg/ml |
| Goat Anti-Rabbit IgG H&L (Alexa Fluor 594) (Abcam, UK) | 20 μg/ml |

*Derivation of LOs from hiBCs after electroporation and transduction*

For obtaining LOs from hiBC line №4 7 days after transgene injection, cells were harvested with the Versene solution, counted using a Countess II FL Automated Cell Counter (Thermo Fisher Scientific, USA), and centrifuged at 150× g for 5 min. The pellet was resuspended in undiluted cold Matrigel at a concentration of 400–1000 cells/μL and replated in 20 μL drops into the wells of a 48-well plate (Corning, USA). The drops were allowed to solidify for 40 min in an incubator, after which the SFDM supplemented with 10 ng/ml FGF7, 10 ng/ml FGF10, 10 ng/ml EGF, 3 µM CHIR99021, and 10 μM Y-27632 was added. After 24 h, the medium was replaced with the Y-27632-free medium.

***Results***

For quantitatively and qualitatively assess the percentage of hiBCs 48 h after transduction was conducted immunofluorescence staining on two basal cell markers (TP63 and KRT5) and on antibody by transgenes (GFP and mCherry). The visualization of immunofluorescence staining is shown in Supplementary Figure 2. The average percentage of TP63+ cells 98.8 ± 0.04% and KRT5+ cells 98.8 ± 0.01% (SD). All cells stained for basal cell markers (TP63 and KRT) at 48 hours after transduction, that demonstrated preservation of basal cell phenotype.

**
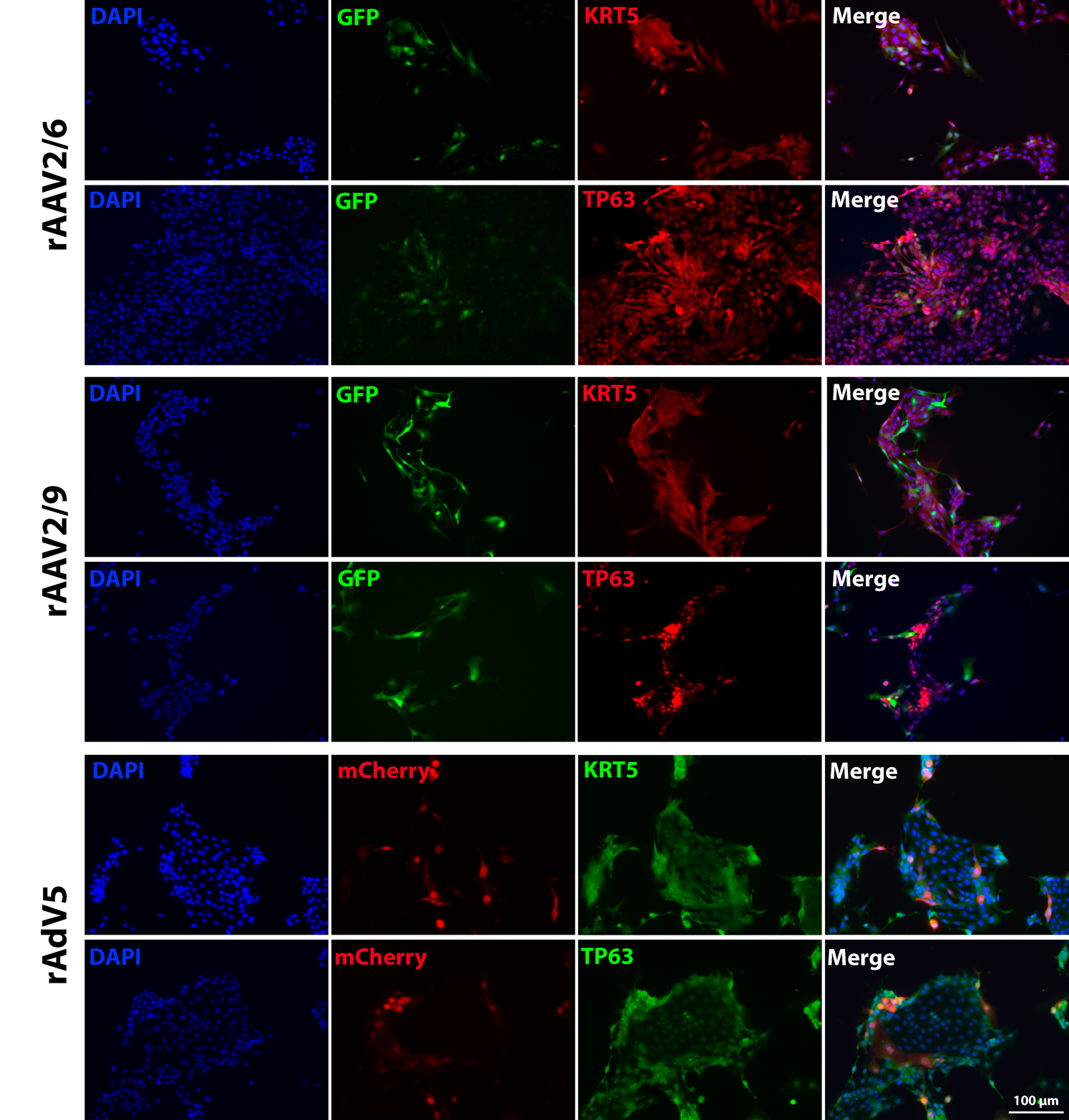
Supplementary Figure 2.** Representative images from fluorescent microscopy of hiBC line №4, stained against major basal cell markers and antibodies by transgenes, 48 hours after transduction. Scale bar, 100 μm

Supplementary Figure 3 demonstrates images of hiBC at 2 and 7 days after transgene delivery. There are no visual differences in cell morphology when compared to the control group. Additionally, hiBCs 7 days after transgene injection successfully form lung organoids (Supplementary Figure 3), demonstrating the preservation of differentiation potential by hiBCs after transgene injection.


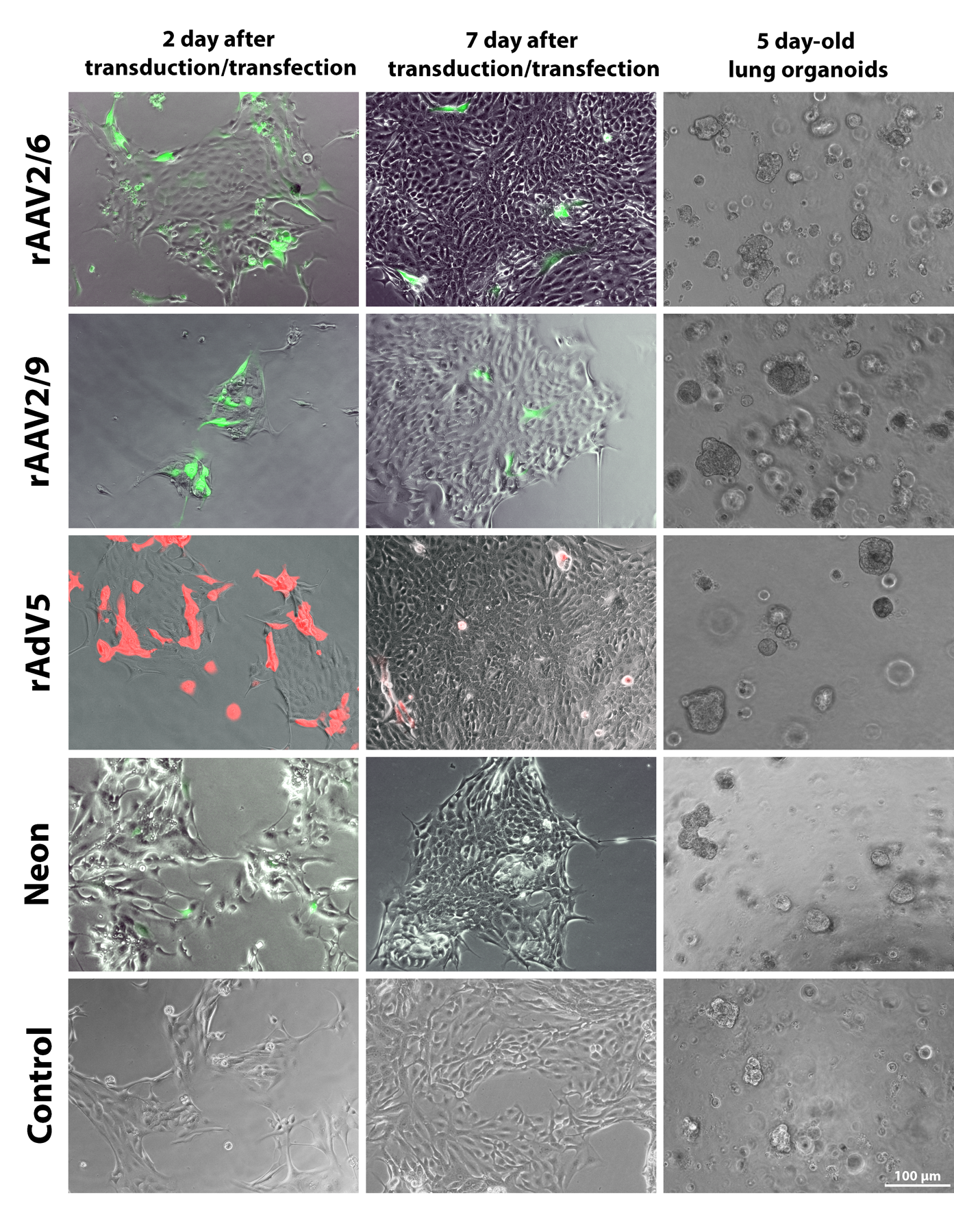


**Supplementary Figure 3.** Merge images from fluorescent and phase-contrast microscopy of hiBCs at 2 and 7 days after transgene delivery compare to control group and phase-contrast microscopy of lung organoids derived from hiBCs at 7 days after transgene delivery. Scale bar, 100 μm
