# Supplementary figures and images for: Airway basal cells from human-induced pluripotent stem cells: a new frontier in cystic fibrosis research
Source: Front Cell Dev Biol. 2024 Apr 26;12:1336392. doi: 10.3389/fcell.2024.1336392 (PMC11082282; doi:10.3389/fcell.2024.1336392)

**$\beta$ -tubulin**

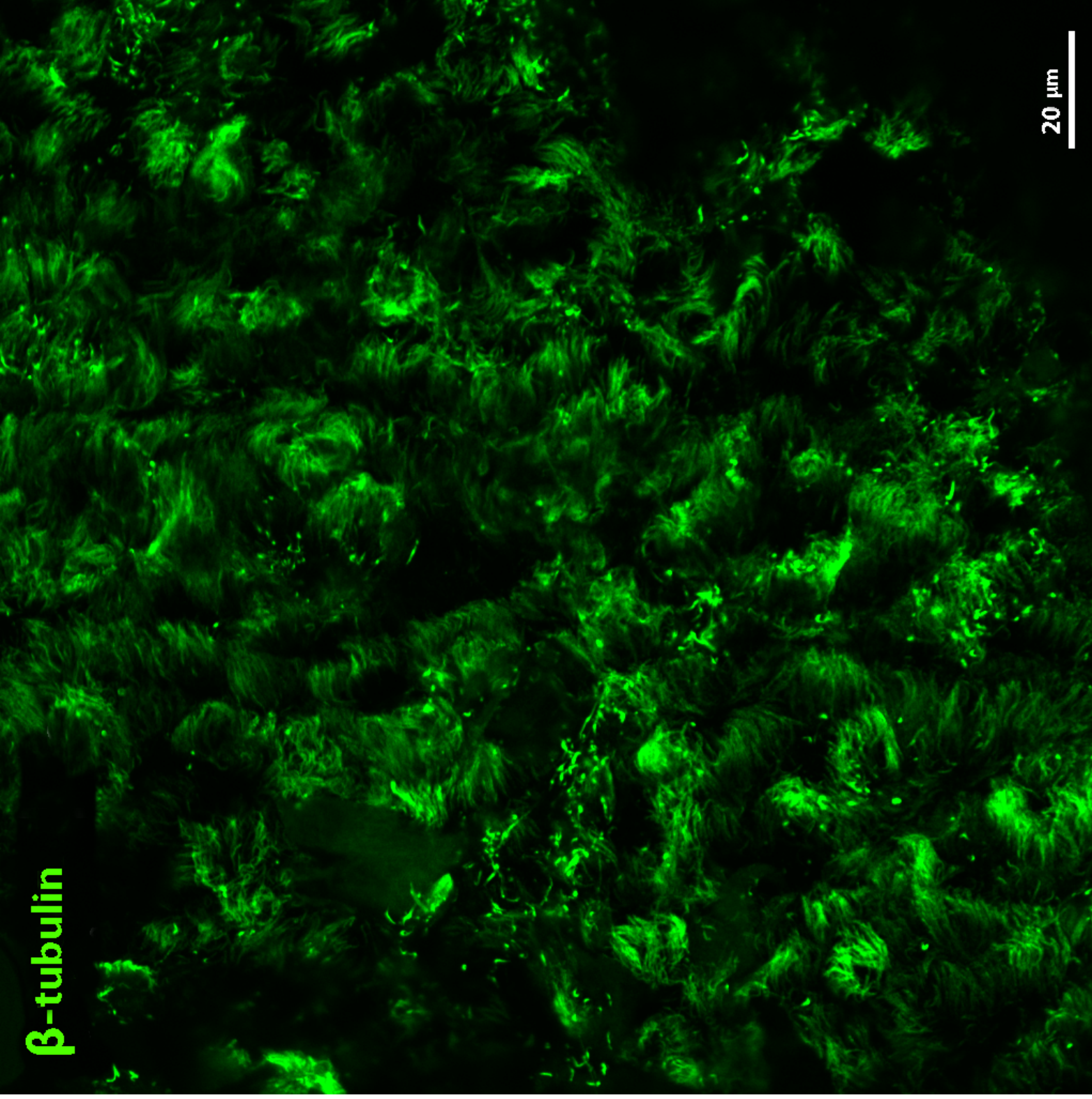

20  $\mu$ m

Supplement: Supplementary file 2 [file DataSheet1.PDF]
